# The complete mitochondrial genome of middle-sized form of *Sthenoteuthis oualaniensis* (Cephalopoda: Ommastrephidae) from the South China Sea

**DOI:** 10.1080/23802359.2020.1797562

**Published:** 2020-07-28

**Authors:** Lei Xu, Xuehui Wang, Delian Huang, Yafang Li, Lianggen Wang, Jiajia Ning, Feiyan Du

**Affiliations:** a Guangdong Provincial Key Laboratory of Fishery Ecology and Environment, South China Sea Fisheries Research Institute, Chinese Academy of Fishery Sciences, Guangzhou, China; b Key Laboratory of South China Sea Fishery Resources Development and Utilization, Ministry of Agriculture, Guangzhou, China

**Keywords:** Mitochondrtal genome, *Sthenoteuthis oualaniensis*, the South China Sea

## Abstract

The purpleback flying squid (*Sthenoteuthis oualaniensis*) is a pelagic squid with tremendous potential for commercial exploitation. *Sthenoteuthis oualaniensis* comprises two forms in the South China Sea, medium-sized form and dwarf form. In this study, we described the complete mitochondrial genome of medium-sized form of *S. oualaniensis.* The genome is 20,309 bp in length, encoding the standard set of 13 protein-coding genes, 20 tRNA genes, and two rRNA genes, with circular organization. The overall base composition of the whole mitochondrial genome was A (35.86%), T (33.36%), G (11.63%), and C (19.15%) with an AT bias of 69.22%. The longest protein-coding gene of these species was *ND5*, whereas the shortest *ATP8*.

The purpleback flying squid (*Sthenoteuthis oualaniensis*; Lesson, 1830) is widely distributed in the equatorial and sub-tropical areas of the Pacific and the Indian Oceans (Voss 1973), it does not extend into the temperate Pacific, and however, it is most abundant in the South China Sea and north-western Indian Ocean (Nesis 1977; Mohamed et al. 2006). This squid is a species of growing commercial interest (Zuyev et al. 2002; Chen et al. 2007) and of special interest from the viewpoint of effective and rational use of the world ocean’s biological resources (Trotsenko and Pinchukov 1994). Voss (1973) speculated a potential of the purpleback flying squid of at least 100,000 metric tons in the Central Eastern Pacific. It is on record that the purpleback flying squid is caught commercially in the eastern and southern East China Sea, Taiwan to Okinawa by hook and line with light at night (Tung 1981; Okutani and Tung 1987). In recent years, because of the drastic decline in traditional fishery resources in the South China Sea, the focus of marine exploitation has shifted to *S. oualaniensis* due to its high biomass, short life cycle, high growth rate, and high fecundity (Chen et al. 2007; Zhang et al. 2010).

*Sthenoteuthis oualaniensis* comprises multiple forms (Nesis 1993; Dunning 1998), varying both in size at maturity and in the possession of a distinctive large dorsal photophore. A middle-sized ‘typical’ form with the photophore is found throughout the species’ range in the eastern tropical Pacific. Equatorial waters of the Indian and Pacific Oceans are inhabited by an ‘early-maturing’ dwarf form that lacks the dorsal photophore and may constitute a separate, as yet undescribed, species (Nesis 1993; Staaf et al. 2010). The study of the population genetic structure of *S. oualaniensis* can provide essential information for the better management and sustainable utilization of this species. Therefore, more molecular data and genome study for this species still welcome, in spite of various molecular markers such as microsatellites and specific COI primer set were developed in this species, recently (Lin et al. 2015; Xu et al. 2017).

The specimens of medium-sized form ‘typical’ of *S. oualaniensis* collected from the South China Sea (17°59′N, 111°59′E) on 7 April 2017. Whole genomic DNA was extracted from muscle tissue of one specimen of *S. oualaniensis* using TIANamp Marine Animals DNA Kit (TIANGEN, China). The concentration for use as a PCR template was adjusted to an A_260_ of about 0.05–0.2. The collected specimen and extracted DNA were stored in Guangdong Provincial Key Laboratory of Fishery Ecology and Environment (specimen accession number: SCS2017-S7-315). The complete mitochondrial genomes of middle-sized form of *S. oualaniensis* was sequenced using PCR primers designed from highly conserved regions of transfer RNA (tRNA) sequences of related species (Yokobori et al. 2004) with additional specific primers designed as required from sequences already obtained. The COI sequence of middle-sized form of *S. oualaniensis* was used as reference seeds for iterative assembly by MITObim v.1.8 (Hahn et al. 2013). SeqMan v.7.1.0 was used for the mitogenome assembly and annotation (Swindell and Plasterer 1997). Transfer RNA genes were predicted using online software tRNAScan-SE 1.21 (Lowe and Eddy 1997). All Protein-coding genes (PCGs) are aligned independently, then concatenated to be applied for phylogenetic reconstruction with other cephalopods in MrBayes v 3.12 (Ronquist and Huelsenbeck 2003) using a relaxed clock model.

The medium-sized form of *S. oualaniensis* mitochondrial genome forms a 20,309 bp closed loop (GenBank accession number MT661575). The overall base composition of the whole mitochondrial genome was A (35.86%), T (33.36%), G (11.63%), and C (19.15%) with an AT bias of 69.22%. This mitochondrial genome represents a typical cephalopods mitochondrial genome and matches with the *Dosidicus gigas* genome, in which it comprises 13 protein-coding genes, 20 transfer RNA genes, two rRNA genes (12S rRNA and 16S rRNA), and one A + T-rich region which could also be termed as control region. The ATG initiation codon is used in all protein-coding genes except *ND1* (ATA), *ND2* (ATT), and *ND5* (ATT), and the stop codons of all the 13 protein-coding genes were complete. Six protein-coding genes (*ATP8*, *ND3*, *COX2*, *COX3*, *ND2*, and *ND5*) use TAA as the termination codon; seven protein-coding genes (*ND4L*, *ND6*, *ATP6*, *ND1*, *CYTB*, *ND4*, and *COX1*) use TAG as the termination codon. Meanwhile, the longest protein-coding gene of these species was *ND5* (1710 bp), whereas the shortest *ATP8* (156 bp). *lrRNA* and *srRNA* genes are 1299 bp and 914 bp in length separately. All the 20 typical tRNAs possess a complete clover leaf secondary structure, ranging from 64 bp to 80 bp. The Bayesian inference phylogenetic tree showed that medium-sized form of *S. oualaniensis* firstly grouped with the dwarf form of *S. oualaniensis* from the eastern Pacific, and closely related to *Dosidicus gigas* (Figure 1). Phylogenetic analyses consistent with the classification results depended on the morphological characters. We have the confidence to construct phylogenetic trees, based on the complete the mitochondrial genomes, but the evolution history of cephalopods still needs future research to be clearly resolved.

**Figure 1.**
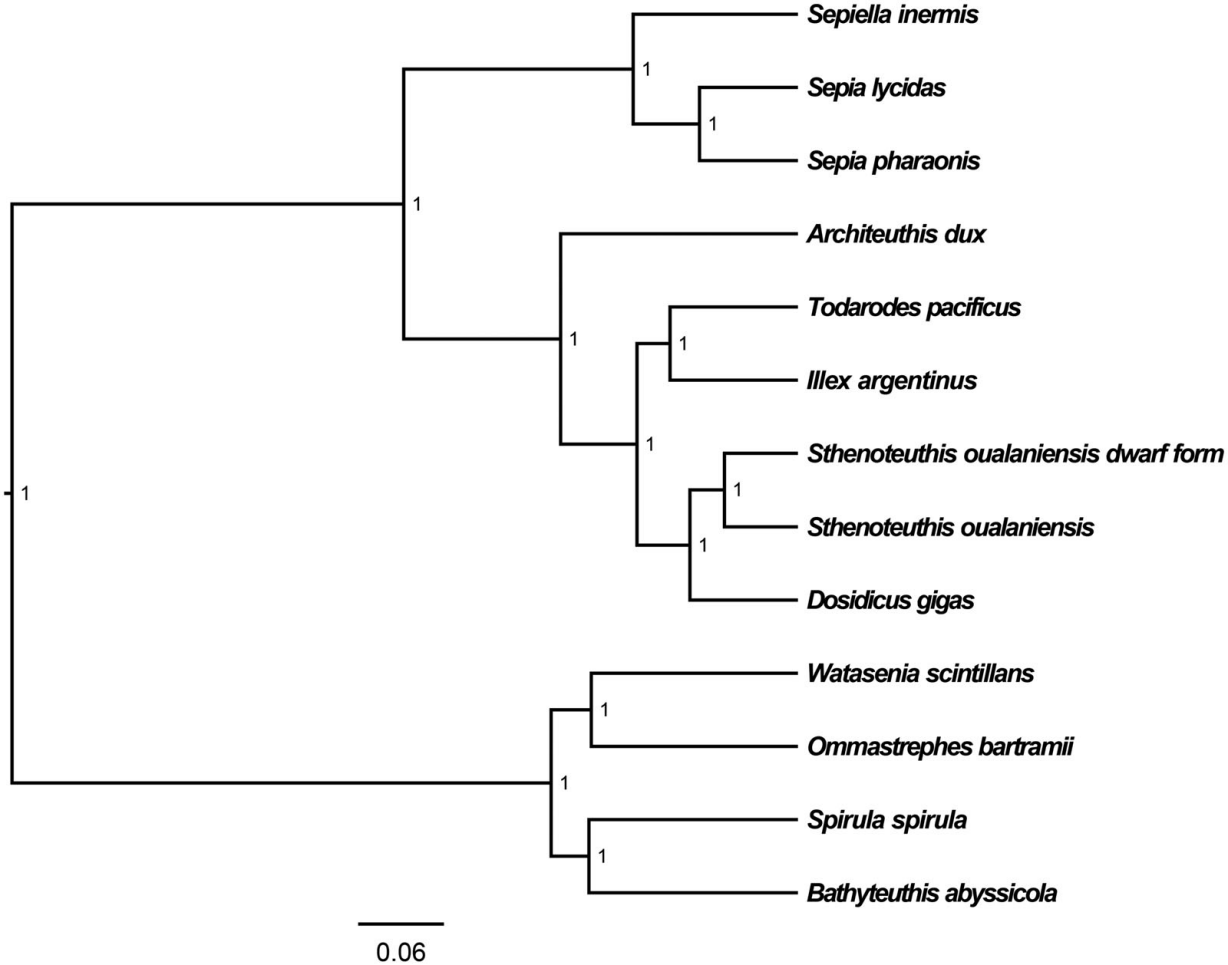
The Bayesian inference phylogenetic tree for Cephalopoda based on mitochondrial PCGs and rRNAs concatenated dataset. The gene’s accession numbers for tree construction are listed as follows: *Sthenoteuthis oualaniensis* from eastern Pacific (EU660577), *Dosidicus gigas* (EU068697), *Todarodes pacificus* (AB158364), *Illex argentinus* (KP336702), *Watasenia scintillans* (AB086202), *Ommastrephes bartramii* (AB715401), *Spirula spirula* (KU893141), *Architeuthis dux* (KC701734), *Bathyteuthis abyssicola* (AP012225), *Sepiella inermis* (KF040369), *Sepia lycidas* (KJ162574), and *Sepia pharaonis* (KC632521).

## Data Availability

The data that support the findings of this study are openly available in GenBank of NCBI at https://www.ncbi.nlm.nih.gov, reference number MT661575.
